# The Effect of Dietary Advice Aimed at Increasing Protein Intake on Oral Health and Oral Microbiota in Older Adults: A Randomized Controlled Trial

**DOI:** 10.3390/nu15214567

**Published:** 2023-10-27

**Authors:** Kristina S. Fluitman, Tim van den Broek, Ilse Reinders, Hanneke A. H. Wijnhoven, Max Nieuwdorp, Marjolein Visser, Richard G. IJzerman, Bart J. F. Keijser

**Affiliations:** 1 Department of Internal Medicine, Amsterdam University Medical Centers, Location VUmc, 1081 HV Amsterdam, The Netherlands; 2 Amsterdam Public Health Research Institute, 1081 HV Amsterdam, The Netherlands; 3 Department of Microbiology and Systems Biology, TNO Earth, Life and Social Sciences, 3704 HE Zeist, The Netherlands; 4 Department of Health Sciences, Faculty of Science, Vrije Universiteit Amsterdam, 1081 HV Amsterdam, The Netherlands; 5 Department of Vascular Medicine, Amsterdam University Medical Centers, Location AMC, 1105 AZ Amsterdam, The Netherlands; 6 Department of Preventive Dentistry, Academic Center for Dentistry Amsterdam, University of Amsterdam and VU University, 1081 LA Amsterdam, The Netherlands

**Keywords:** oral microbiota, oral health, protein intake, older adults

## Abstract

Nutrition and oral health are closely related, especially in older adults in whom poor nutrition may lead to oral microbial perturbations, exacerbating poor oral health. In a 6-month randomized controlled trial, we evaluated the effects on oral microbiota and on oral health of dietary advice aimed at increasing protein intake to ≥1.2 g/kg adjusted body weight/day (g/kg aBW/d) in community-dwelling older adults with low habitual protein intake (<1.0 g/kg aBW/d). Food intake was measured via 24 h dietary recalls, oral health was measured via questionnaires, and oral microbial composition was assessed via the 16S rRNA sequencing of tongue swabs. Mean baseline protein intake was 0.8 g/kg aBW/day in both groups. In the high protein group (*n* = 47), participants increased their protein intake to mean 1.2 g/kg aBW/day at the 6-month follow-up. Protein intake in the control group (*n* = 43) remained at 0.9 g/kg a BW/day. The intervention did not affect self-reported oral health. While it caused moderate shifts in oral microbiota alpha- and beta-diversity measures, abundances of individual bacterial taxa were not affected. In conclusion, our intervention did not affect self-reported oral health within a period of 6 months, nor did it substantially affect the tongue microbiota composition.

## 1. Introduction

It is well-established that nutrition and oral health are closely related [1]. This relationship is especially evident in older adults, in whom both oral health and nutritional status are often compromised [2,3]. Poor oral health in older adults is characterized by high rates of caries, periodontal disease, edentulism, and xerostomia [3], which are largely microbiota-associated diseases [4]. Several cross-sectional studies have confirmed associations of poor oral health with lower and less varied nutrient intake [5,6,7], particularly a lower intake of protein [8]. Poor oral health is thought to disturb masticatory function, and lead to decreased food intake and protein-energy malnutrition [9]. Vice versa, it has been suggested that insufficient protein intake aggravates poor oral health in various ways [10,11,12], but evidence is scarce.

Protein deficiency is said to compromise the integrity of dentition and its supporting structures. It is also thought to result in delayed wound healing and poor resistance to oral pathogens [10]. Moreover, protein deficiency is said to reduce salivary flow and alter its composition, limiting its protective abilities [12]. Protein-energy malnutrition may also cause salivary gland atrophy and be related to enamel hypoplasia, predisposing individuals to dental caries [1,12]. It must be noted that the studies underpinning these mechanisms focus on children or animal models [12]. There is also new evidence suggesting that protein intake is associated with the composition of the oral microbiota. A recent cross-sectional observational study of 59 healthy adults found that protein intake was positively associated with several bacterial taxa, including Selenomonas, Johnsonella, Prevotella, Peptostreptococcus, and Actinomyces [11]; however, the clinical implications are unclear. The effect of dietary protein on the oral microbiota composition may constitute a novel pathway by which nutrition affects oral health. As of yet, no interventional studies have been performed to evaluate the effects of increasing protein intake on oral health or the oral microbiota in older adults.

Currently, the European Food Safety Authority recommends 0.83 g protein intake per kg body weight per day, irrespective of age [13]. However, many researchers argue that older adults should at least consume 1.0–1.2 g per kg body weight per day, due to higher protein requirements in older adults [14,15]. We conducted a randomized controlled trial on the effects of dietary advice aimed at increasing protein intake to ≥1.2 g/kg adjusted body weight (aBW)/day in older adults with habitual low protein intake (i.e., <1.0 g/kg aBW/d) on physical functioning [16]. In a subgroup of trial participants, we evaluated the effects on oral health and the oral microbiota composition. We hypothesize that the increase in protein intake will improve oral health and alter the oral microbiota.

## 2. Materials and Methods

This study was part of the 6 month, multicenter, randomized controlled PROMISS trial, performed from November 2018 to July 2020 at the University of Helsinki, Finland, and the Vrije Universiteit Amsterdam, the Netherlands [16]. PROMISS’s main objective was examining the (cost-)effectiveness of personalized dietary advice aimed at increasing protein intake to 1.2 g/kg aBW/d during a 6-month period on changes in physical functioning in community-dwelling older adults with a habitual protein intake of <1.0 g/kg aBW/d [16]. The full PROMISS study protocol has been published elsewhere [16]. Moreover, the intervention’s effect on the gut microbiota and appetite has also been published in a prior ancillary study [17]. The present ancillary study evaluates the effect of the intervention on oral health and the oral microbiota composition. All participants provided written informed consent before enrolling in the trial. The trial was registered at ClinicalTrials.gov (NCT03712306). It was approved by both the Institutional Review Boards of the Amsterdam UMC, location VUMC in Amsterdam, the Netherlands (approval code: 2018.399, approval date: 5 June 2019), and by the University of Helsinki (approval code: HUS/1530/2018, approval date: 12 June 2019). It was conducted in accordance with the Declaration of Helsinki (version 2013).

### 2.1. Participants

As reported earlier [16,17], a total of 276 participants were included in the PROMISS main trial. Inclusion criteria for PROMISS were: age ≥ 65 years, community-dwelling, habitual protein intake <1.0 g/kg aBW/d, BMI ≥ 18.5 kg/m^2^ and ≤32.0 kg/m^2^, and ability to walk 400 m within 15 min without the use of a walker and with no rest >60 s. Participants were excluded if they adhered to a vegan diet, had severe food allergies, purposefully lost or gained >3 kg in past 3 months, had diagnosed severe kidney disease, type 1 diabetes or insulin dependent type 2 diabetes, an eating disorder, severe acute heart disease in the past 3 months, or poor cognitive status determined by a mini-mental state examination score ≤ 20 [18]. Additionally, participants were excluded from this ancillary microbiota study if they had inflammatory bowel disease, had been institutionalized (>4 weeks) in the past 3 months, or had used systemic antibiotics in the past 3 months.

PROMISS participants were randomized into 3 groups. One group received dietary advice aimed at increasing protein intake (*n* = 96). One group received dietary advice aimed at increasing protein intake and were advised to consume their protein in close proximity to physical exercise (*n* = 89). And one control group received no intervention (*n* = 91). Participants were stratified based on baseline habitual protein intake (<0.9 or 0.9–1.0 g/kg aBW/d) and sex. For the current microbiota ancillary study, only participants from the first intervention group and the control group were included. At first, only Dutch participants were included in the ancillary microbiota study. However, because of a slow inclusion rate, inclusion was later expanded to the Finnish PROMISS participants as well.

**Figure 1:**
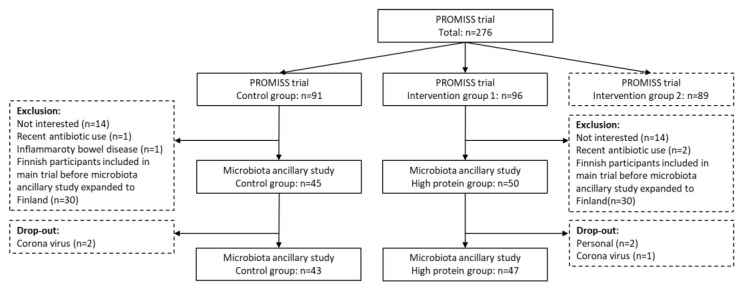
Flowchart of inclusion of the PROMISS microbiota ancillary study.

A total of 90 participants were included, 47 from the high protein group and 43 from the control group (Figure 1).

### 2.2. Intervention

Assessment at the clinic took place at baseline, at 3-month follow-up, and at 6-month follow-up. The high protein group was provided with dietary advice orally and in writing. Patients received individualized advice tailored to their habitual dietary characteristics, body weight, and food preferences [16]. The advice aimed to increase protein intake to ≥1.2 g/kg aBW/d with at least one meal containing ≥35 g protein. We aimed to keep total daily energy intake stable.

Actual body weights were adjusted to the nearest weight that would place a participant in the healthy BMI range. This was performed for those with a BMI of <18.5 kg/m^2^ (age ≤ 70 years)/< 22.0 kg/m^2^ (age > 70 years) or a BMI of 25.0–32.0 kg/m^2^ (age ≤ 70 years)/27.0–32.0 kg/m^2^ (age > 70 years). This adjustment prevented the overestimation or underestimation of the participants’ protein requirements because of increased protein needs (in case of low body weight) or excessive adipose tissue (in case of high body weight). Food intake was measured using food-diary assisted 24-h dietary recalls at baseline and 3- and 6-month follow-up.

### 2.3. Oral Health and Bio-Sampling

Data on oral health were collected using a questionnaire, which inquired about the number of remaining teeth, the frequency of tooth brushing and interdental cleaning, and whether participants had experienced caries, bleeding gums, red or swollen gums, oral blisters or soars, toothache when consuming hot or warm drinks or when chewing, lost or loose or broken teeth, halitosis, or xerostomia in the previous 6 months [19].

Participants were asked not to brush their teeth on the day of oral sampling. Unstimulated salivary flow (g/5 min) was collected in 50 participants according to the method of Navazesh [20]. Data on the oral microbiota composition were collected through the 16S rRNA sequencing of a tongue swab samples. Duplicate tongue swab samples were collected by stroking the posterior tongue dorsum 4 times using a Copan eNAT swab (Copan Italia S.p.A., Bréscia, Italy). After collection, the tongue swabs were stored in eNAT RNA/DNA stabilizing and preservation medium. Tongue swabs were initially stored at −20 °C and moved to central storage at −80 °C once a week. Due to the COVID pandemic, not all 6-month follow-up visits could be performed at the research unit. Because of this, 20 participants in the Netherlands were visited at home for final measurements. In Finland, *n* = 16 participants were sent the questionnaires and materials for tongue swabs and were instructed to fill out the questionnaires and collect the tongue swab samples themselves. The tongue swabs were then kept in their home freezers at −20 °C until all swabs were collected from the participants’ houses and transported to central storage at −80 °C on dry ice. Dates and times of sampling and storage at −20 °C and −80 °C were noted for all samples.

### 2.4. 16S rRNA Sequencing

For the 16S rRNA amplicon sequencing of the V4 hypervariable region, previously described methods were used [21,22]. Each sample was PCR-amplified using 1 ng of template DNA with primers F515/R806, targeting the V4 hypervariable region of the 16S ribosomal gene [23]. A mixed pure culture isolates (mock), Blanco extraction controls, pooled salivary extraction controls, and pooled DNA amplification controls were included in each sample batch. The amount of DNA per sample was quantified using the Quant-iT™ PicoGreen^®^ dsDNA Assay Kit (Thermo Fisher Scientific, Waltham, MA, USA). The amplicon libraries were pooled in equimolar amounts and purified using the IllustraTM GFXTM PCR DNA and Gel Band Purification Kit (GE Healthcare, Eindhoven, The Netherlands). Amplicon quality and size were analyzed on the Fragment Analyzer (Advanced Analytical). The paired-end sequencing of amplicons was conducted through five separate runs on the Illumina MiSeq platform (Illumina, Eindhoven, The Netherlands). The denoising of sequence data and the identification of amplicon sequence variants (ASVs) were performed using the DADA2 (1.12.1) pipeline [24]. The median sequencing depth was 69,250 reads. In total, 2482 ASVs were identified and were matched to existing species from the Human Oral Microbiome Database [25].

### 2.5. Statistics

For non-microbiota data, SPSS software version 22 (SPSS Inc., Chicago, IL, USA) was used. Student’s *t*-test, the Mann–Whitney U-test, and Fisher’s exact test were used to assess between-group baseline characteristics. As reported before [17], mixed effects models were used to test the intervention effect on macronutrient intake over time. Fixed effects were visit (time), group (high protein or control), and baseline values. A random intercept was included to account for repeated measures. If there was a significant difference between groups over time, linear regression analyses were used for the 3- and 6-month follow-ups separately, which was adjusted for baseline values. This was also performed in order to analyze salivary flow rate at the 6-month follow-up. Log transformation was applied in cases of non-parametric distributions skewed to the right. Between-group differences in oral health at the 6-month follow-up were tested with logistic regression adjusted for baseline oral health.

All statistical analyses of the microbiome were carried out using R (version 4.0.3) [26]. Figures were constructed using the ggplot2 package (version 3.2.1) [27]. For all microbiota analyses, except those on alpha-diversity, the microbiota data were filtered to include only those ASVs that contribute to the first 97.5% of all counts in the data, eliminating sparse, low count ASVs from the dataset. Inverse Simpson and Shannon alpha-diversities were calculated using the vegan package (version 2.5–3) [28]. This package was also used to perform multivariate analysis and ordinations, based on Bray–Curtis distances. The multivariate models fitted through PERMANOVA were tested via permutation analysis, using 103 permutations. Linear mixed models from the DESeq2 package (version 1.20.0) and the Dream package (version 1.23.0) were utilized, using the Variance Partition extension [29,30,31] to test for differentially abundant taxa. The Benjamini–Hochberg correction for multiple testing was applied. A *p*-value <0.05 was considered statistically significant.

## 3. Results

As reported before [17], the high protein and control groups in our microbiota subsample of PROMISS trial participants did not differ based on age, sex, MMSE-score, or level of education. Nor did they differ based on oral hygiene routines. All baseline characteristics are depicted in Table 1.

### 3.1. Dietary Intake

There were no baseline between-group differences in energy and macronutrient intake (Table 1). In our subpopulation, participants from the high protein group increased their mean protein intake from 0.8 ± 0.2 g/kg aBW/d at baseline to 1.3 ± 0.3 g/kg aBW/d at 3-month follow-up and 1.2 ± 0.2 g/kg aBW/d at 6-month follow-up. In contrast, the average protein intake for the control group remained at 0.9 ± 0.2 g/kg aBW/d. These differences in protein intake were statistically significant based on linear mixed models (*p* < 0.0001) and linear regression analysis for each time point (B = 0.4, *p* < 0.001; B = 0.3, *p* < 0.001, for the 3- and 6-month follow-ups, respectively) [17]. Daily energy intake also differed between groups over time: 1873.9 ± 454.2 kcal/d and 1836.1 ± 382.9 kcal/d at 3- and 6-month follow-ups in the high protein group, compared to 1679.2 ± 429.8 kcal/d and 1699.2 ± 341.6 kcal/d at 3- and 6-month follow-ups in the control group (*p* = 0.0008). This was also true for carbohydrate intake: 187.7 ± 51.0 g/d and 185.0 ± 48.9 g/d at 3- and 6-month follow-ups in the high protein group versus 177.0 ± 54.6 g/d and 174.2 ± 47.7 g/d at 3- and 6-month follow-ups in the control group (*p* = 0.0367). However, using linear regression models, no significant differences were found in either energy intake or carbohydrate intake for each time point separately. Fat intake remained unaffected by the intervention. The results of the analyses on dietary intake are described more elaborately elsewhere [17].

### 3.2. Oral Health

There were no baseline differences in dentition, frequency of tooth brushing, or frequency of interdental cleaning (use of floss, toothpicks, or interdental brushes) (Table 1). The intervention did not decrease the frequency with which participants reported caries; bleeding gums; red or swollen gums; blisters or soars; tooth ache when drinking hot or cold drinks or when chewing; lost, loose, or broken teeth; halitosis; or xerostomia at the 6-month follow-up (Table 2). It also did not affect salivary flow rate (B = 1.2, *p* = 0.165).

### 3.3. Oral Microbiota

**Figure 2:**
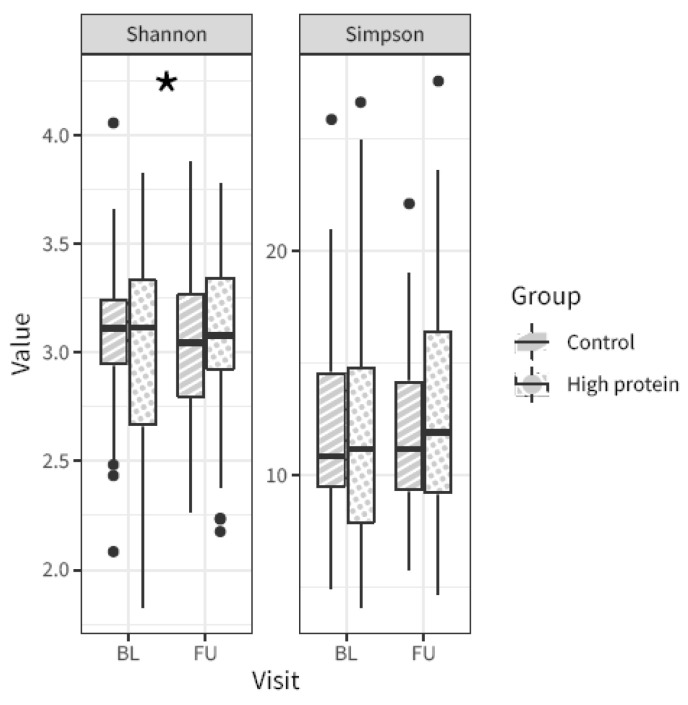
Microbiota alpha-diversity from baseline (BL) to follow-up (FU) in the high protein compared to control group, based on the Shannon index and the Simpson index. Asterisk indicates *p*-value linear mixed model visit × intervention < 0.05. Boxplot center lines indicate median, boxes indicate interquartile ranges, whiskers indicate 1.5× the interquartile ranges and black dots indicate outliers.

Alpha-diversity (i.e., the microbial diversity of a sample) was calculated using the Shannon and Simpson indices. The Shannon alpha diversity index showed a slight, albeit significant difference in change from baseline to follow-up between the high protein and control group (linear mixed model visit × intervention for the Shannon and Simpson alpha diversity measures *p* = 0.015 and 0.133, respectively). In particular, the control group showed a slight decrease in alpha-diversity from baseline to follow-up, whereas the high protein group stayed unchanged. There was no difference in the Simpson index (Figure 2).

**Figure 3:**
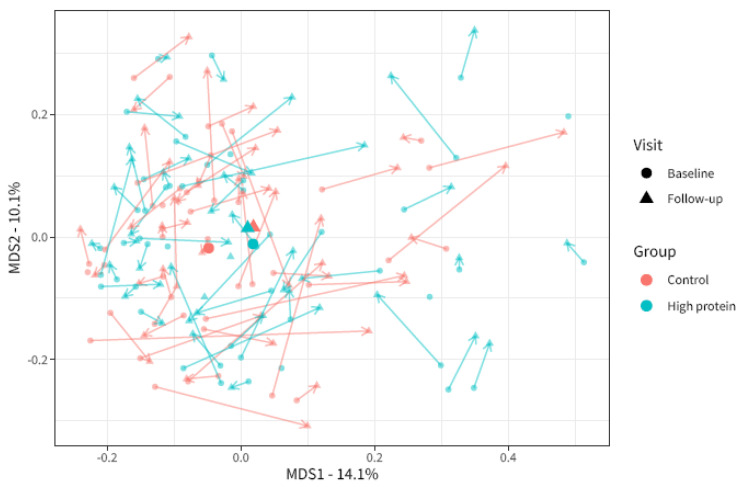
Multi-dimensional scaling plot of microbiota beta-diversity based on Bray–Curtis dissimilarity for baseline and follow-up visits in the high protein and control groups. Each point indicates one sample from one participant. The larger points depict the centroids. The axis depicts 14.1% and 10.1% of variation in Bray–Curtis dissimilarity in the study population. The closer the points to each other, the more similar the microbial composition. Baseline (BL) and follow-up (FU) samples from the same participants are linked by arrows.

Beta-diversity (i.e., the microbial dissimilarity between samples) was assessed using the Bray–Curtis dissimilarity measure. This was used to evaluate the overall microbial compositional differences between groups. Bray–Curtis dissimilarity changed significantly from baseline to follow-up in the high protein group compared to the control group (Figure 3, PERMANOVA analyses visit × intervention R^2^ = 0.004, *p* = 0.013). In other words, the increased protein intake caused a slight shift in the overall microbial community composition compared to control. However, we were not able to identify any individual microbial taxa that were specifically affected by the intervention.

## 4. Discussion

Our study demonstrates that increasing protein intake from an average of 0.8 g/kg aBW/d to 1.2 g/kg aBW/d does not affect self-reported oral health status in older adults. Moreover, whereas moderate effects were observed on the overall microbiota composition based on alpha- and beta-diversity measures, no individual bacterial taxa were found to be specifically affected.

To our knowledge, we were the first to study the effects of increasing protein intake on either oral health or the oral microbiota composition in older adults. Although protein deficiencies have previously been suggested to negatively impact oral health [1,10,12], increasing protein intake did not improve self-reported oral health in our study. Importantly, even though our participants had low protein intake at baseline, they were not necessarily protein deficient. Possibly, the oral health benefits of increasing dietary protein only occur in cases of more severe protein deficiency. Moreover, our mean increase in protein intake may have been too modest, and the six-month follow-up period too short for our intervention to effectuate a detectable change in some aspects of oral health.

As for the effect of protein intake on the oral microbiota, some studies have demonstrated periodontitis-associated bacterial communities to display the elevated expression of proteolytic genes [32]. This raises the concern that increasing protein intake may increase the substrate for these dysbiotic communities and promote their growth. However, this concern is not substantiated by our results as no specific species were affected by the intervention. Contrary to our results, Cattaneo et al. [11] showed that several bacterial taxa from the tongue dorsum were associated with protein intake, suggesting that altering protein intake could potentially affect these taxa. There are several explanations as to why we could not demonstrate this. First, the associations found by Cattaneo et al. were cross-sectional, based on pre-existing dietary patterns in young adults. The dietary patterns in these participants may have been shaping their oral microbiota for many years. In contrast, our dietary intervention was implemented for 6 months, prior to which our groups had similar intakes of protein and other macronutrients. Second, compared to other body sites, oral microbiota display high levels of intra-individual (alpha) diversity and temporal stability [33,34]. As a result, the oral microbiota may be resilient to dietary interventions.

### Strengths and Limitations

To ensure adherence and an accurate increase in protein intake, nutritionists tailored their dietary advice to suit each participant’s personal eating behaviors and preferences. Moreover, throughout the study, participants provided the nutritionists with feedback on the feasibility of their protein-enriched diets, allowing the nutritionists to adjust their dietary advice accordingly. Indeed, the advice succeeded in increasing the average protein intake to 1.2 g/kg aBW/d. Even though the increase in protein intake may have been too moderate to influence oral health or the oral microbiota in a meaningful way, the intervention was in line with the increased recommended daily allowance for protein intake in older adults suggested by expert groups [14,15]. An important limitation is that oral health was assessed by means of questionnaires, rather than oral examination. Possibly, the protein-effect on oral health was not reflected by an altered frequency of self-reported oral discomfort but could have been observed by oral exams. However, self-perceived oral health and clinically determined oral health were previously found to be closely associated [35,36]. Second, microbiota composition was only sampled on the tongue, whereas this constitutes only one of several microbial niches within the oral cavity, each with its own distinct bacterial community [33]. Our protein intervention did not affect the specific taxa of the tongue microbiota but may have affected taxa from other oral niches, such as dental plaque. However, obtaining a standardized plaque sample in older adults is difficult due to the prevalent use of dentures. Additionally, in a previous study, the tongue microbiota were found to be relevant to oral health in community-dwelling older adults [37].

## 5. Conclusions

Here, we demonstrate that dietary advice aimed at increasing protein intake to at least 1.2 g/kg aBW/d in older adults with habitual low protein intake did not significantly improve self-reported oral health. Although our intervention moderately affected overall microbiota composition (expressed in alpha- and beta-diversity measures), no individual taxa were significantly increased or decreased. A future study implementing a more drastic or more prolonged increase in dietary protein in older adults with more severe protein deficiencies may show a more pronounced effect on oral health or the oral microbiota.

## Figures and Tables

**Table 1:** Baseline characteristics of microbiota subsamples.

	High Protein Group (*n* = 47)	Control Group (*n* = 43)	*p*-Value
Characteristics			
Age (years)	74.6 ± 4.8	74.1 ± 4.7	0.572
Sex (Male)	28 (59.6)	19 (44.2)	0.205
BMI (kg/m^2^)	26.1 ± 2.9	26.8 ± 2.9	0.227
MMSE	29 (27–30)	29 (27–30)	0.573
Education			0.11
Low	3 (6.4)	0 (0.0)	
Middle	8 (17.0)	13 (30.2)	
High	36 (76.6)	30 (69.8)	
Study site (Amsterdam)	35 (74.5)	33 (76.7)	1
Time to follow-up (days)	182 (178–187)	185 (178–191)	0.187
Oral Hygiene			
Dentition			0.842
No teeth	5 (10.9)	6 (14.0)	
Some teeth	7 (15.2)	8 (18.6)	
Most to all teeth	34 (73.9)	29 (67.4)	
Teeth brushing			0.698
<2×/day	17 (36.2)	12 (27.9)	
2×/day	24 (51.1)	25 (58.1)	
>2×/day	6 (12.8)	6 (14.0)	
Interdental cleaning			0.802
Never	7 (14.9)	6 (14.0)	
sporadic	15 (31.9)	11 (25.6)	
regularly	25 (53.2)	26 (60.5)	
Food intake			
Energy intake (kcal/day)	1701.9 ± 427.4	1611.0 ± 301.8	0.244
Protein intake (g/kg aBW/day)	0.8 ± 0.2	0.8 ± 0.1	0.689
Protein intake (g/day)	62.9 ± 14.0	60.9 ± 10.4	0.457
Carbohydrate intake (g/day)	181.4 ± 56.4	170.9 ± 47.6	0.347
Fat intake (g/day)	67.3 ± 20.4	66.1 ± 18.3	0.771

BMI: Body mass index; MMSE: mini-mental state exam; aBW: adjusted body weight. Values are depicted as mean ± standard deviation (parametric continuous), median (interquartile range) (non-parametric continuous), or number (%) (categorical). Baseline differences between groups were tested via the independent sample *t*-test (parametric continuous), independent sample Mann–Whitney U-test (non-parametric continuous), or Fisher’s exact test (categorical).

**Table 2:** Intervention effect on self-reported oral health.

	High Protein Group (*n* = 47)		Control Group (*n* = 43)		Differences at Follow-Up
	Baseline	6 Months	Baseline	6 Months	OR (95%CI)
Any oral discomfort (yes/no)	26 (55.3)	31 (66.0)	35 (81.4)	34 (79.1)	0.9 (0.3–3.0)
Caries	5 (10.6)	5 (10.6)	6 (14.0)	6 (14.0)	0.8 (0.2–2.9)
Bleeding gums	4 (8.5)	7 (14.9)	7 (16.3)	4 (9.3)	3.7 (0.7–21.2)
Red/swollen gums	4 (8.5)	4 (8.5)	6 (14.0)	4 (9.3)	1.0 (0.2–4.3)
Blisters/soars	5 (10.6)	6 (12.8)	2 (4.7)	6 (14.0)	0.7 (0.2–2.5)
Toothache—hot/cold	4 (8.5)	2 (4.3)	4 (9.3)	4 (9.3)	0.4 (0.1–2.6)
Toothache—chewing	1 (2.1)	2 (4.3)	0 (0.0)	3 (7.0)	0.6 (0.1–3.8)
Lost/loose/broken teeth	7 (14.9)	7 (14.9)	5 (11.6)	4 (9.3)	1.6 (0.4–6.3)
Halitosis	4 (8.7)	4 (8.5)	5 (11.6)	7 (16.3)	0.5 (0.1–2.2)
Xerostomia	14 (30.4)	17 (36.2)	21 (48.8)	17 (39.5)	1.6 (0.5–5.3)

Shown are the numbers (and percentages) and odds ratios (95% confidence intervals). Differences between groups were tested with logistic regression for follow-up values, adjusted for baseline.

## Data Availability

These clinical trial data can be requested by any qualified researcher who engage in rigorous, independent scientific research, and will be provided upon reasonable request by the corresponding author. The 16S rRNA sequencing data have been deposited in the NCBI Sequence Read Archive (SRA) under accession number PRJNA1029554.
